# Proportion of doctors who stayed in the state of Tocantins after finishing medical residency: preliminary results from a cross-sectional study

**DOI:** 10.1590/1516-3180.2016.0340280117

**Published:** 2017-07-31

**Authors:** Leonardo Baldaçara, Raquel Prudente de Carvalho Baldaçara

**Affiliations:** I MD, PhD. Assistant Professor, Universidade Federal do Tocantins (UFT), Health Department of the State of Tocantins, Palmas (TO), Brazil.

**Keywords:** Internship and residency, Education, medical, Specialization, Employment, Physicians

## Abstract

**CONTEXT AND OBJECTIVE:**

Few studies have assessed the impact of medical residencies on the public healthcare system. The aim here was to assess the number of specialists who remained in the state of Tocantins after finishing the medical residency program during the first two years of the first programs (2013 and 2014).

**DESIGN AND SETTING:**

Cross-sectional and exploratory study conducted at the Federal University of Tocantins in Brazil.

**METHODS:**

All graduates of medical residency programs in Tocantins, of the years 2013 and 2014, were interviewed by telephone and e-mail between May and July 2014.

**RESULTS:**

Information was obtained from 37 graduates from medical residency. Seventeen (50.0%) were working in the state public healthcare system and only six (17.6%) in a municipal service in June 2014. Considering only the 24 doctors who had never worked in the state of Tocantins before their residency, it was observed that two who graduated in 2013 (20.0%) and five who graduated in 2014 (35.7%), i.e. seven out of the total number (29.2%), had established their homes in Tocantins.

**CONCLUSIONS:**

The number of graduates from medical residency who stayed in the state of Tocantins in 2013 and 2014 was small. However, this was related to the absence of other programs for continuation of the specialization. The state healthcare system was primarily responsible for employment of these doctors within public services. On the other hand, hiring by municipal services was extremely low.

## INTRODUCTION

The 2011 Medical Demographic Census in Brazil reported that 45% of Brazilian physicians were not specialists.^1^ Only 1.9% of medical residency positions and 3.47% of the specialists were located in northern Brazil. Thus, in the state of Tocantins, a lack of professionals working in the public healthcare system was identified, as also in the whole Legal Amazon region.^1^ Through the National Program to Support Training of Medical Specialists in Strategic Areas (“Pró-residência”),^2^ the first medical residencies were created in this state. The project was supported by the supposition that specialists were more likely to establish themselves in places where they had trained. However, there is no consensus that installation of medical courses (undergraduate or postgraduate level) can allocate new professionals.

The 2013 Medical Demographic Census in Brazil reported that almost two-thirds of doctors remained in the place where they graduated and about a third returned to their hometowns.^3^ Regardless of whether professionals graduated in the major centers, these places have greater weight in determining whether doctors stay in the same location.^3^

Interestingly, only a few papers on this topic have been published.^1^^,^^3^ It is very important to assess the impact of implementing new medical residency programs in priority regions, in order to ascertain whether this type of training stimulates greater numbers of professionals to establish themselves in these locations. Therefore, since Tocantins was the last Brazilian state to open residency programs and there is a national task force to open such postgraduate programs, it is an ideal place to conduct such research.

## OBJECTIVE

The aim of this study was to assess the proportion of doctors who stayed in the state of Tocantins after finishing medical residency, after the implementation of the first programs.

## METHODS

This was an observational and exploratory cross-sectional study that assessed doctors who finished medical residency programs in the state of Tocantins in the years of 2013 and 2014. Those who did not agree to participate and those who could not be located were excluded. The interviews took place between May and July 2014, which was the fourth year in which the programs had been implemented.

The variables (questions) related to the following: age, gender, medical residency field and year of graduation from residency; whether the subjects were currently (after graduation from residency) working in the state of Tocantins, and if not, why not; whether they were working in a municipal public service; whether they were working in a state public service; whether they were working in the private system in Tocantins; whether they had worked during the residency to supplement their income; current income; and whether before or during the medical residency they had been working in a state or municipal public service.

The interviews took place via email. Subjects who did not respond were contacted by telephone. Among the 37 graduates from residency, 34 participated in the survey. This study was submitted to and approved by the Research Ethics Committee of the Federal University of Tocantins.

### Statistical analysis

The data were recorded and analyzed through the IBM SPSS 22.0 software. The variables were presented as absolute numbers and proportions. Subsequently, a comparative analysis was performed using the chi-square test. The significance (α) value selected was ≤ 0.05.

## RESULTS

Over the period of this study, the Federal University of Tocantins offered 28 vacancies for medical residency each year: 6 in internal medicine, 6 in general surgery, 6 in pediatrics, 4 in gynecology and obstetrics, 5 in family and community medicine and 1 in psychiatry. However, the occupation rate of these vacancies was low. Over these two years, there were only 37 graduates from 51 vacancies, thus corresponding to an occupancy rate of 72.5%. Information was obtained from 34 of the 37 graduates, i.e. 3 were excluded from the analysis because they could not be contacted. For more details, see Table 1.

**Table 1.** Status of graduates from medical residency in the state of Tocantins in July 2014

Variable	2013 n = 15	2014 n = 19	Total n = 34
Age	31 ± 3	30 ± 3	30.2 ± 3.4
Male	8 (50.0%)	7 (33.3%)	15 (40.5%)
Program			
Internal medicine	8 (50.0%)	8 (38.1%)	16 (43.2%)
General surgery	3 (18.8%)	3 (14.3%)	6 (16.2%)
Gynecology and obstetrics	0	2 (9.5%)	2 (5.4%)
Pediatrics	5 (31.3%)	6 (28.6%)	11 (29.7%)
Family and communitymedicine	0	1 (4.8%)	1 (2.7%)
Psychiatry	0	1 (4.8%)	1 (2.7%)
Working in the state of Tocantins			
State network	7 (46.7%)	10 (52.6%)	17 (50.0%)
Municipal network	3 (20.0%)	3 (15.8%)	6 (17.6%)
Private network	7 (46.7%)	10 (52.6%)	17 (50.0%)
The current salary is higher than before and/or during residence	4 (26.7%)	7 (36.8%)	11 (32.4%)
Doctors who were already civil servants in Tocantins before medical residency	5 (33.3%)	5 (73.7%)	10 (29.4%)
Doctors who were new to Tocantins and stayed in the state	2 (20.0%) (n = 10)	5 (35.7%) (n = 14)	7 (29.2%) n = 24)

### Employment situation in June 2014

Regarding the employment situation in June 2014, after finishing medical residency, 17 (50.0%) were working in the state of Tocantins and had established their homes there. In comparing the graduates from the years 2013 (7; 46.7%) and 2014 (10; 52.6%), it was observed that the increase in establishment rate was low and not significant (X^2^ = 0.119; P = 0.500). All the specialties were represented among the doctors who had established their homes in Tocantins.

Seventeen (50.0%) were working in the state public healthcare system: seven (46.7%) who had finished their medical residency in 2013 and ten (52.6%) in 2014 (X^2^ = 0.119; P = 0.500). All the specialties were represented among the doctors who were working in this type of system.

However, only six (17.6%) were working in a municipal public healthcare system: three (20.0%) from 2013 and three (15.8%) from 2014 (X^2^ = 0.102; P = 0.749). The specialties of this doctors were: internal medicine, general surgery, pediatrics and community and family medicine. No doctors who had finished residencies were working in the specialties of obstetrics and gynecology or psychiatry, in any municipal network.

The reasons reported by the graduates for not remaining in the state were that they had started attending another residency and that the salary offered was low, compared with other states.

Finally, since these were the first medical residency programs in the state of Tocantins, the subjects were asked whether they were already public employees of the state and whether, through the creation of new courses, they had had the opportunity to specialize. In other words, it was assessed whether the trained physicians were already living and working in the state before the medical residency program. It was found that ten (29.4%) were already working in the state of Tocantins and were civil servants. After finishing the medical residency program, they continued in their jobs. A new analysis was then carried out, only among the doctors who had never worked in the state of Tocantins. Out of these 24 subjects, 2 from 2013 (20.0%) and 5 from 2014 (35.7%), i.e. 7 (29.2%) out of the total number were working in the state in June 2014 (X^2^ = 8.697; P = 0.404). In other words, the capacity of medical residency to lead to establishment of new specialists over the short term was low.

Only 4 (26.7%) from 2013, 7 (36.8%) from 2014 and 11 (32.4%) from the total number reported that their current salary was higher than before or during the medical residence.

## DISCUSSION

Although Brazil has a national rate of 2.11 doctors per 1,000 inhabitants, the inequalities in the distribution of doctors are enormous. Inequalities exist between different federal states, between state capitals and elsewhere in the respective state, and between groups of municipalities stratified according to population. Data from 2014 showed that northern Brazil (1.09 physician per 1,000 inhabitants) and northeastern Brazil (ratio of 1.3) were below the national rate. However, the highest proportion of doctors per capita (1.51/1,000 inhabitants) in these regions was recorded in Tocantins.^4^ Throughout the country, areas away from larger cities show the greatest unevenness of coverage and lack of attendance.^4^ Our study corroborates this, in that we noted that most of the specialists who settled in Tocantins stayed in the capital. Even when they went elsewhere in the state, they remained in larger cities.

In previously published data, from 107,114 doctors who graduated from residency in places differing from where they were born, 27,106 (25.31%) were living in the city where they graduated. Among these were some major centers of attraction: about 60% of those who stayed where they graduated from residency remained in seven state capitals, of which five were in southeastern Brazil.^3^ The other 40,618 (37.92%), who graduated from residency in locations other than where they were born, were now practicing their activity and/or living in another place, i.e. differing both from where they were born and from where they graduated from residency.^3^ In our study, we initially observed an establishment rate of 50%. However, after excluding the confounding factor of already having gained admission as a civil servant in the state of Tocantins before entering the residency, this figure fell to 29.2%.

According to the 2015 census, 41% of physicians in Brazil did not have any specialty. In the country as a whole, the ratio was 1.41 specialists for each generalist.^4^ The South had the highest proportion of specialists (2.11) in relation to generalists.^4^ At the other extreme, the northern region had the lowest proportion (0.94), thus indicating that for every specialist there was a generalist. In Tocantins, this ratio was 0.80 specialist for each generalist.^4^

In the same way as shown in data from a previous study,^5^ the present study showed that there was little demand for and training of specialists in family and community medicine. On the other hand, there was a larger number of trainees within pediatrics.

A previous study^6^ that was conducted to identify the characteristics of Memorial University of Newfoundland medical graduates and the predictors that they would continue working in Canada and Newfoundland and Labrador (NL) after residency training concluded that the medical school had made a substantial contribution to the local supply of physicians, such that it had produced over half of the physicians working in that province in 2004. The authors proposed initiatives to increase national and provincial retention of medical graduates: attracting rural students to medical careers, increasing admission of local students and providing incentives for graduates to complete their residency training in the province.^6^ In an another study from the same team, it was observed that medical graduates originating from other countries were less likely to work in Canada and NL.^7^ On the other hand, another study investigated the factors that family physicians reported as influencing their recruitment and retention. These authors observed that family doctors’ decisions to settle and remain in a community were multifactorial. They proposed that the community’s educational system and the spouse’s needs would have to be directly addressed in order to ensure effective recruitment and retention.^8^ A further study assessed factors relating to career longevity that were reported by residency-trained emergency physicians: interactions with residents, higher income, satisfaction with training decisions and board certification in emergency medicine were variables associated with a higher retention rate.^9^

One important question is how to attract doctors to work in primary care and in rural areas. No exact answers have been presented, but the following have been found to be predictive of continued retention in the face of adversity: revision of medical school curricula to encompass current inpatient-oriented training programs;^10^ hiring of doctors with residency in family or community practice;^11^^,^^12^ working in a greater city area;^12^ satisfaction with community health centers;^12^ and prior resilience under adverse circumstances.^13^

Landry et al.^14^ observed that medical training at the undergraduate level did not affect the likelihood of ever or currently practicing in a Canadian province. However, doctors who had been exposed to a training program during postgraduate residency were 5.9 times (95% confidence interval, CI: 2.3-14.9) and 3.2 times (95% CI: 0.9-11.6) more likely, respectively, to practice in the province than were doctors without postgraduate exposure.^14^ Their findings were corroborated by Savageau et al.^12^

In Brazil, a previous study identified the following reasons for difficulty in hiring medical specialists, as pointed out by managers of municipal and state public healthcare systems: lack of graduate professionals, according to the criteria of the Ministry of Education and the Brazilian Medical Association; lack of professionals with the experience required for the work; and the fact that professionals consider that the remuneration levels offered by institutions are low.^15^ Comparison between these data and the preliminary results from the present study showed that training more doctors does not lead to more specialists working in the public healthcare system.

We noted that in Tocantins, the main recruitment level was the state, while there was negligible recruitment at municipal level. Among the reasons for difficulties in recruitment given in the abovementioned study,^15^ the main reason why these professionals were not working in municipal healthcare services was probably the remuneration, since the state had more attractive proposals. On the other hand, it is possible to speculate that other factors influence professionals’ interest: working structure, housing structure and urban facilities.

In a previous study, it was found that few doctors remained in the municipal primary care network and that one of the factors for this was the high turnover of professionals.^16^ The municipal administration seemed to contribute directly and strongly towards this phenomenon, as determined by the municipal health bureau. Another 18% was contributed through relationship problems, largely arising from conflicts with management.^16^ Improvement in establishing doctors within primary care can be achieved through resolving difficulties relating to the type of employment contract, career plan or lack thereof, remuneration, working hours and questions of political interference.^16^ Another author proposed a number of measures to assist in securing doctors within the public system, including a career plan for healthcare professionals and federal employment relationships, which also supports the idea that municipalities have great difficulty in hiring such professionals.^17^

It could be seen that in Tocantins, the medical residency program did not have the capacity to ensure that specialists became established in this state over the short term. We also noticed that there was greater fragility in municipal networks, especially outside of the main urban areas. However, it is important to note that the low attachment rate was attributed to the absence of programs for continuing medical education. In the literature, it has been shown that multiple factors, including education, healthcare system administration priority, workplace conditions, family, income, professional self-interest, and resilience can be related to greater retention of doctors after graduation and medical residency.

## CONCLUSION

The number of graduates from medical residency who stayed in the state of Tocantins in the first two years of the program was small, but was related to the absence of other programs for sub-specialization. The state network was primarily responsible for employment of these doctors within the public service. On the other hand, hiring by municipal networks was extremely low. A future survey, after five years of implementation of these programs, will review these rates and assess the impact of creation of new medical residency programs.

Finally, it can be assumed that, without an effective state policy for presence in the economic and social development of underserved areas, and without a policy placing greater value on professionals, so as to attract them to public-service careers, the increasing number of doctors will further increase the inequalities.
